# Ionic Homeostasis Maintenance in ALS: Focus on New Therapeutic Targets

**DOI:** 10.3389/fnins.2018.00510

**Published:** 2018-08-07

**Authors:** Rossana Sirabella, Valeria Valsecchi, Serenella Anzilotti, Ornella Cuomo, Antonio Vinciguerra, Pasquale Cepparulo, Paola Brancaccio, Natascia Guida, Nicolas Blondeau, Lorella M. T. Canzoniero, Cristina Franco, Salvatore Amoroso, Lucio Annunziato, Giuseppe Pignataro

**Affiliations:** ^1^Division of Pharmacology, Department of Neuroscience, School of Medicine, University of Naples Federico II, Naples, Italy; ^2^Centre National de la Recherche Scientifique, Institut de Pharmacologie Moléculaire et Cellulaire, Université Côte d'Azur, Valbonne, France; ^3^IRCCS SDN Napoli, Naples, Italy; ^4^Division of Pharmacology, Department of Science and Technology, University of Sannio, Benevento, Italy; ^5^Department of Neuroscience, Università Politecnica delle Marche, Ancona, Italy

**Keywords:** ionic homeostasis, ALS, neurodegeneration, transporters, channels

## Abstract

Amyotrophic lateral sclerosis (ALS) is one of the most threatening neurodegenerative disease since it causes muscular paralysis for the loss of Motor Neurons in the spinal cord, brainstem and motor cortex. Up until now, no effective pharmacological treatment is available. Two forms of ALS have been described so far: 90% of the cases presents the sporadic form (sALS) whereas the remaining 10% of the cases displays the familiar form (fALS). Approximately 20% of fALS is associated with inherited mutations in the Cu, Zn-superoxide dismutase 1 (SOD1) gene. In the last decade, ionic homeostasis dysregulation has been proposed as the main trigger of the pathological cascade that brings to motor-neurons loss. In the light of these premises, the present review will analyze the involvement in ALS pathophysiology of the most well studied metal ions, i.e., calcium, sodium, iron, copper and zinc, with particular focus to the role of ionic channels and transporters able to contribute in the regulation of ionic homeostasis, in order to propose new putative molecular targets for future therapeutic strategies to ameliorate the progression of this devastating neurodegenerative disease.

## Introduction

Amyotrophic lateral sclerosis (ALS) is a progressive and devastating neurological disease characterized by the loss of Motor Neurons (MNs) in spinal cord, motor cortex and brainstem (Tokuda and Furukawa, 2016). Usually, the disease shows a peak of onset around 45–60 years and has a post-diagnosis survival time of approximately 3–5 years. Nonetheless, ALS is a clinically heterogeneous pathology and some patients survive longer and reveal a less aggressive disease (Brooks et al., 2000; Hilton et al., 2015). Clinically, the loss of motor neurons causes a progressive muscle weakening and fasciculation. In the later disease stages, the patients become paralyzed. In addition, almost half of patients can evidence signs of cognitive impairment and mild memory decline. Ultimately, ALS induces muscle paralysis, respiratory breakdown and early death (Lomen-Hoerth et al., 2003; Ringholz et al., 2005; Rusina et al., 2010).

The neuropathological hallmarks of this neuromuscular disorder are degeneration of MNs in the spinal anterior horn and motor cortex and loss of axons in the lateral columns of the spinal cord (Saberi et al., 2015).

On the basis of the inheritance of the disease, ALS is classified in two forms: the sporadic form (sALS) that includes the majority of ALS cases and, the familiar form (fALS) that regards approximately 5–10% of cases (Katsuno et al., 2012; Wen et al., 2017).

**Figure 1:**
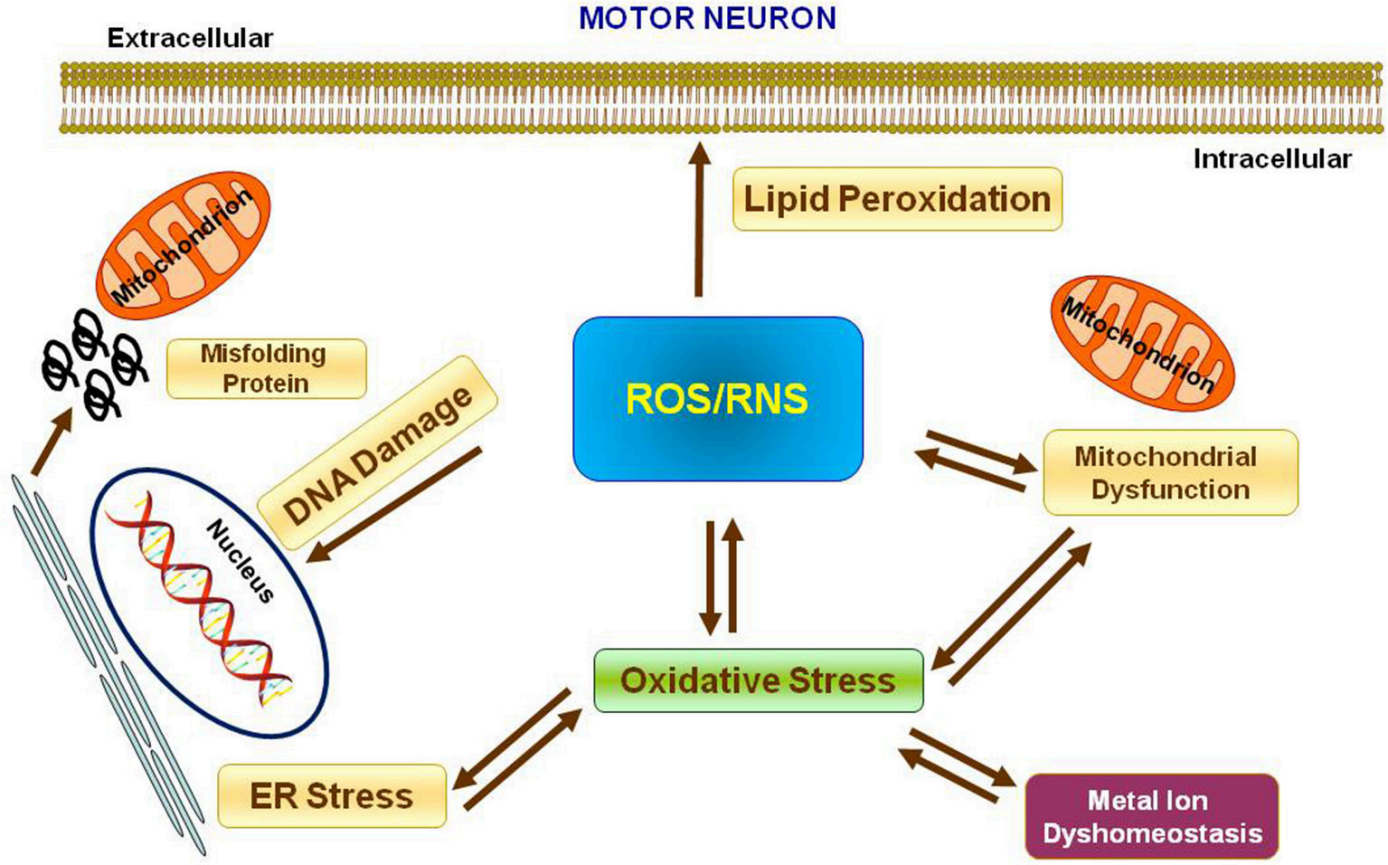
Oxidative stress in Motor Neuron. Scheme representing the production of Reactive Oxygen Species (ROS) and Reactive Nitrogen Species (RNS) and their effects on nucleus, mitochondria, endoplasmatic reticulum and lipid peroxidation.

Metal ions are essential cofactors for enzymes and structural elements for stabilizing static biomolecules (Que et al., 2008). They also participate to brain metabolism by controlling neurotransmitter synthesis, nerve transition, and oxygen transport (Crichton et al., 2008). Most importantly, metal ions may take part to the generation of oxidative stress. Indeed, hyperproduction of reactive oxygen species (ROS) and reactive nitrogen species (RNS) is due to either metal ion dyshomeostasis or imbalance between the generation of free radicals and their destruction by antioxidants, leading to cellular damage, aging, and apoptosis through oxidation of principal cellular components (i.e., lipids, proteins, and DNA). It is not yet established whether metal interaction represents initial or secondary factor, or whether it represents a consequence of the neurodegeneration (Gilgun-Sherki et al., 2001; Carrí et al., 2003; Chen et al., 2003; Visconti et al., 2005; Crichton et al., 2008; Valko et al., 2016; Sheykhansari et al., 2018). Mitochondria are the main site of ROS production and cells apoptosis. They are vulnerable to ROS and it has been confirmed that mitochondrial injury intensifies ROS and oxidative damage in several neurodegenerative disorders including ALS (Figure 1).

**Figure 2:**
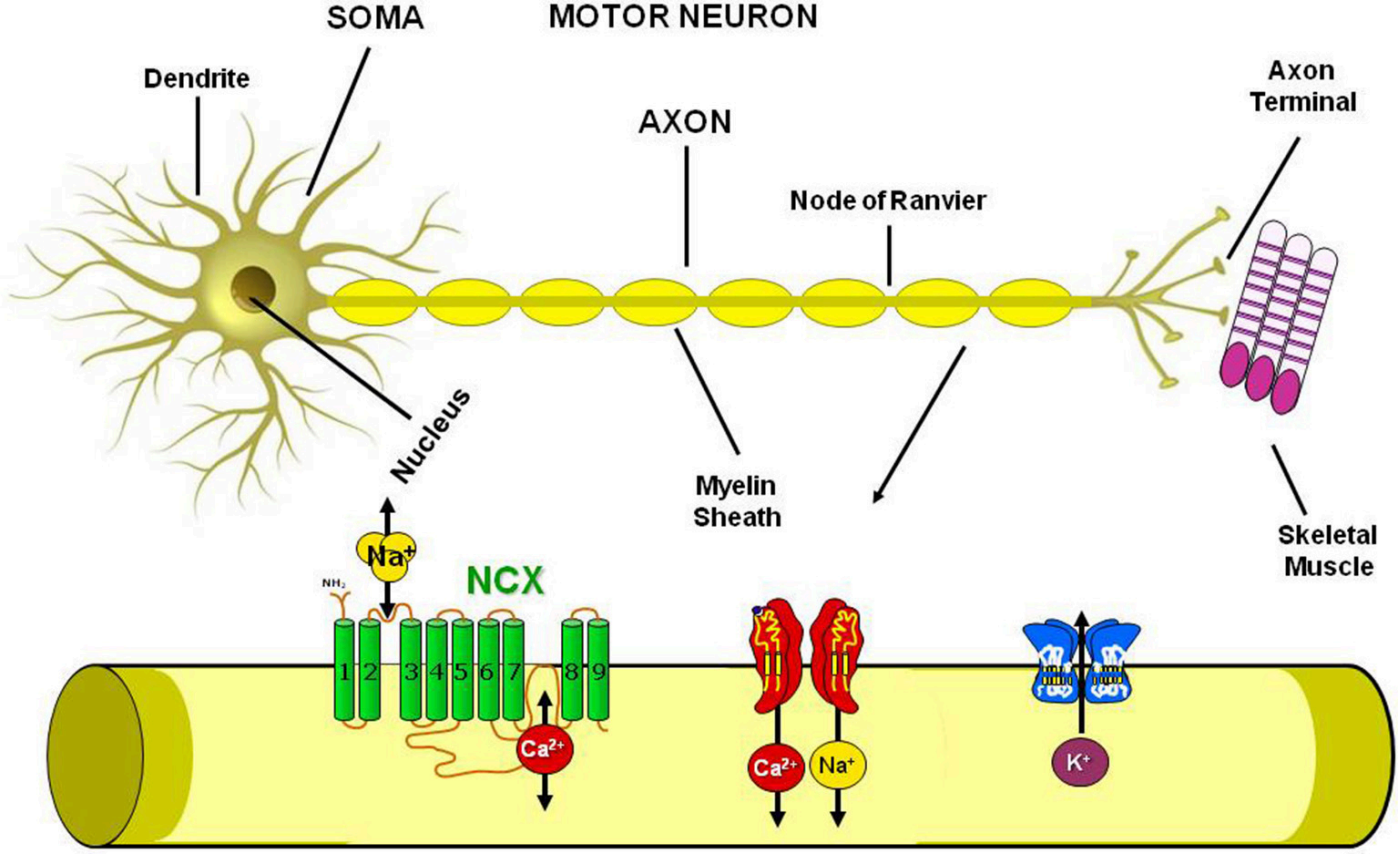
Plasmamembrane ion channels and transporters in the Motor Neuron. Scheme representing the axon structure and the distribution in the motor neuron plasmamembrane of Na^+^/Ca^2+^ exchanger, Na^+^, Ca^2+^ and K^+^ channels.

Standing on these premises and since ionic homeostasis dysregulation has been assumed to represent one of the main trigger of the pathological cascade that culminates in MNs loss, in the present paper we will review the involvement in ALS pathophysiology of the most well studied metal ions, i.e., calcium, sodium, iron, copper and zinc, with particular regards to the possible role of ionic transporters and channels involved in the regulation of ionic homeostasis as putative molecular targets for future therapeutic strategies aiming to reduce the progression of this devastating neurodegenerative disease (Figure 2).

## Role of calcium (Ca^2+^) IONS

Defects in cellular Ca^2+^ signaling are involved in the pathogenesis of many neurodegenerative diseases, including ALS, as widely reported in literature (Siklós et al., 1998; Jaiswal, 2013; Mühling et al., 2014). Calcium is one of the most important intracellular messenger, being involved in neuronal development, synaptic transmission and plasticity, as well as in the regulation of several metabolic central nervous system (CNS) pathways (Grosskreutz et al., 2010). Indeed, combining the lessons from multiple *in vivo* and *in vitro* models used to determine ALS pathogenic mechanisms, what comes out is that a combination of mechanisms involved in mitochondrial dysfunction, ER stress and Ca^2+^ homeostasis maintenance may be responsible for the vulnerability of MNs observed in ALS (Jaiswal and Keller, 2009; Jaiswal, 2013; Petrozziello et al., 2017). In this regard, several evidence support the idea that deregulation of glutamate neurotransmission by increasing extracellular glutamate levels, probably for the oxidative damage to the excitatory amino acid transporter-2 (EAAT2) that reduced glial glutamate uptake (Lin et al., 2012), may trigger Ca^2+^ entry, finally leading to altered Ca^2+^ homeostasis crucial for MN degeneration (Plaitakis and Caroscio, 1987; Rothstein et al., 1992, 1995; Couratier et al., 1993; Trotti et al., 1999, 2001). In fact, it has been verified that ROS generated in MNs may cross the plasmamembrane and damage glutamate transporters in neighboring astrocytes. Notably, studies carried out in cell lines and animal mouse models demonstrate that these mechanisms are common to both the sporadic and the familiar forms of ALS (Jaiswal, 2013). Further, Ca^2+^ buffering proteins (CaBPs) such as calbindin and parvalbumin (Alexianu et al., 1994; Palecek et al., 1999; Jaiswal, 2013) are lost at an early stage of the disease in hypoglossal, spinal and low cranial MN populations (Ferrer et al., 1993) thus suggesting the association of intracellular Ca^2+^ homeostasis disturbance in ALS progression (Siklós et al., 1998; Grosskreutz et al., 2010; Jaiswal, 2013; Mühling et al., 2014).

In line with this evidence, since low cytosolic Ca^2+^ buffering ability represents a main risk factor for degeneration, an increase in cytosolic Ca^2+^ buffering capacity protects vulnerable MNs from degeneration (von Lewinski and Keller, 2005). Motor neurons express several Ca^2+^ channels, either activated by ligand or voltage, able to mediate fast Ca^2+^ entrance which, in turn, leads to the impairment of some mechanisms of extrusion such as the plasmamembrane calcium ATPase and the Na^+^/Ca^2+^ exchanger (NCX), due to the quite weak cytosolic Ca^2+^ buffering property of these transporters, resulting in an excess of mitochondrial Ca^2+^ and ROS production. Moreover, the entry of Ca^2+^ into the mitochondria contributes to establish the chronic depolarization of the mitochondrial membrane, which consequently determines the release of pro-apoptotic proteins and the activation of enzymes able to activate other cell death pathways (Jaiswal, 2013). Hereupon, we documented the importance of the plasmamembrane Na^+^/Ca^2+^ exchanger isoform 3 (NCX3) in ALS pathogenesis because animals affected by ALS show a strong reduction in its expression and activity at muscular and neuronal levels, whereas strategies able to delay ALS progression worked also through NCX3 activation and overexpression (Anzilotti et al., 2018). NCX, by contributing to the maintenance of Na^+^ and Ca^2+^ homeostasis, takes part to the progression of some neurological diseases including stroke, seizure, multiple sclerosis and Alzheimer Disease (Annunziato et al., 2004, 2007, 2013; Sirabella et al., 2009; Molinaro et al., 2011, 2013; Pannaccione et al., 2012; Lanzillotta et al., 2013). Up to now, within the CNS, three different isoforms, NCX1, NCX2 and, NCX3, and numerous splicing variants have been identified; the precise involvement of each NCX isoform in ALS progression and etiology has not yet been determined, nonetheless some seminal works postulated a crucial role for NCX3 in mediating the impairment in neuromuscular transmission occurring ALS and in other related disease (Sokolow et al., 2004; Boscia et al., 2012; Casamassa et al., 2016; Anzilotti et al., 2018), thus rendering it a putative druggable target in ALS. On the other hand, as previously mentioned, In familiar ALS, mitochondrial Ca^2+^ overload, caused by Cu/Zn-superoxide dismutase 1 (SOD1) mutation, causes strong ROS generation. In these patients, riluzole, a drug able to increase survival rate, has shown to prevent MNs deterioration and moderately reduce excitotoxicity and cell loss by blocking tetrodotoxin-sensitive sodium channels which are associated with damaged neurons. Deregulation of intracellular calcium homeostasis has also been described in the terminal motor axons of subjects affected by ALS, as well as in the spinal MNs of ALS animals, both in the sporadic and familial forms (Jaiswal, 2017). Recently, an additional possible mechanism of action has been reported for riluzole. This involves a considerable reduction of [Ca^2+^]_i_ transient currents and a reversible inhibition of [Ca^2+^]_i_ inward currents in MNs of adult symptomatic SOD1 G93A mice (Jaiswal, 2017); thus confirming the importance of Ca^2+^ homeostasis in ALS pathophysiology.

## Role of sodium (Na^+^) and potassium (K^+^) IONS

In sporadic amyotrophic lateral sclerosis phenotypes alterations of axonal excitability, associated to increased of persistent sodium (Na^+^) conductance and reduced potassium (K^+^) currents, have been described and related to the evolution of ALS signs and symptoms such as neurodegeneration and fasciculation. In fact, membrane hyperexcitability observed in ALS due to Na^+^ and K^+^ conductances abnormalities, leads to muscle cramps and fasciculations, and promotes a neurodegenerative cascade mediated by Ca^2+^-dipendent processes. In addition, modulation of axonal Na^+^ channel function in ALS resulted in amelioration of symptoms and stabilization of axonal excitability parameters. Axonal ion channel dysfunction evolves with disease progression and correlates with survival, thus representing a potential therapeutic biomarker in ALS (Park et al., 2017). In this context, in sASL axonal degeneration has been linked to upregulation of persistent Na^+^ conductances. Moreover Na^+^ conductance increase has also been reported in transgenic SOD1 mice although mechanisms of ectopic activity, such as cramp and fasciculations, and axonal degeneration still necessitate clarifications in patients affected of familiar ALS (fALS), and, specifically, it is not clear whether any difference occurs with the processes identified in subjects affected by the sporadic form of ALS (sALS). Furthermore, several studies documented a deregulation of voltage-dependent Na^+^ currents (Nav) and increased persistent sodium current (PICNa) in primary neuronal cultures obtained from ALS mice. At a molecular level, voltage-gated Na^+^ channels are composed by a single 260 kDa α subunit and one or more β subunits of 30–40 kDa. The α subunits constitute the actual ion channel and include sensors for voltage dependence. These subunits represent the working cores of the excitation process, whereas β subunits control the kinetics of channel activation and inactivation in dependence from the voltage and the localization of the channel on the plasmamembrane (Catterall, 2014; Chen-Izu et al., 2015; Kubat Öktem et al., 2016).

Expression of Nav1.3 appears very early in embryogenesis and reaches a maximum level of expression at birth then it starts reducing after the second postnatal week, reaching very low levels at maturity age. Beside Nav1.3, the other Na^+^ channel isoform most expressed in MNs during embryonic development is Nav1.2 (Beckh et al., 1989; Goldin, 1999; Alessandri-Haber et al., 2002). By contrast, the expression levels of other two Na^+^ channels isoforms, Nav1.1 and 1.6, are high at later developmental stages (Alessandri-Haber et al., 2002) as evidenced in the rat, where these two isoforms are expressed at high levels in the CNS of adult animals while Nav1.3 levels are substantially reduced (Goldin, 1999). Notably, it has been shown that in the most common mutation occurring in ALS patients, SOD1A4V a shift and increase of total Na^+^ currents voltage dependent by Nav1.3 channel occurs (Alessandri-Haber et al., 2002; Kubat Öktem et al., 2016). In fact, this channel mediates a persistent inward sodium current and has been implicated in human neurological disease (Lampert et al., 2006; Holland et al., 2008). These results suggest that modifications in the biophysical properties of voltage-gated sodium channels are essential in the genesis of mutant SOD1-induced hyperexcitability in ALS (Kubat Öktem et al., 2016).

As documented also by a recent study, an overload of Na^+^ and Ca^2+^ ions induced by veratridine combined with TDP-43 overexpression increases early apoptosis of NSC-34 cells and may represent a valid *in vitro* model of ALS involvement of sodium homeostasis-deregulation in ALS (Mouhid Al-Achbili et al., 2016).

As regard a possible role for potassium homeostasis, a recent paper by Bataveljić demonstrated that the impaired ability of astrocytes to preserve water and potassium homeostasis may affect the blood brain barrier (BBB) integrity, may alter the neuronal microenvironment, and may cause motoneuronal dysfunction and death (Bataveljić et al., 2012). Indeed, an increased expression and activity of aquaporin-4 (AQP4) and a decreased expression and activity of inwardly rectifying K^+^ channel (Kir4.1) in the brainstem and cortex of ALS rats and in cultured ALS cortical astrocytes occurs. Since these channels are required for the maintenance of a functional BBB astrocytic lining these results strongly suggest a role for the control of water and K^+^ homeostasis in the disturbance of motor neuron survival during ALS (Bataveljić et al., 2012).

## Role of copper (Cu)

Copper is an essential trace element, playing an indispensable role in the physiology of the human CNS and its intracellular levels are finely regulated (Lutsenko et al., 2010). Increased copper concentrations in the CNS has been observed in patients with neurological symptoms related to Alzheimer's-like dementia and other neurological diseases (Basun et al., 1991). Therefore, alterations in intracellular copper ion homeostasis could represent a possible mechanism responsible for the pathogenesis of ALS (Tokuda et al., 2013). Indeed, in the spinal cord of transgenic rodents carrying different SOD1 mutations the total amount of copper ions has been demonstrated to be anormally elevated in regardless if the mutation affected or not the copper binding affinity of the enzyme (Tokuda et al., 2009, 2013). Interestingly, the levels of other metals, proposed as possible toxic factors in ALS, such as magnesium, aluminum, calcium, manganese and iron, did not change in the spinal cords of the different mouse strains. Moreover, copper dyshomeostasis is evident in G93A SOD1 mice before the onset of clinical symptoms, in a pre-symptomatic phase of the disease, suggesting its increase as a pathological hallmark of the pathology (Tokuda et al., 2013).

Notably, SOD1 different mutants shift the copper trafficking system toward copper accumulation. In particular, the expression levels of the Cu importer 1 (CTR1) and the Cu efflux pump (ATP7A) increases and decreases, respectively, in the spinal cord of mutant animals. Furthermore, also the expression level of metallothioneins (MT), a class of proteins with a very high affinity for copper, is augmented in the spinal cord of G93A SOD1 mice (Gong and Elliott, 2000).

Copper accumulation might be extremely detrimental for the cells. In fact, copper ions in the cuprous state (Cu^+^) can induce oxidative stress directly catalyzing the formation of strong oxidant such as lipid hydroperoxides from hydrogen peroxide and hydroperoxides via a Fenton-like reaction (Halliwell, 2006). Copper is also capable of causing DNA strand breaks and oxidation of bases via ROS. Secondly, exposure to elevated levels of copper significantly decreases glutathione (GSH) levels (Speisky et al., 2009). GSH is a powerful antioxidant, that acts as substrate for several enzymes that remove ROS. It can directly chelate copper, maintaining it in a reduced state. However, elevated cellular Cu levels may deplete glutathione levels, shifting the redox balance toward oxidizing environments, and hence, both enhancing the cytotoxic effect of ROS and allowing the metal to be more catalytically active, thus producing higher levels of ROS (Jomova and Valko, 2011). Furthermore, copper ions have been shown to act as cofactor of pro-inflammatory cytokines in several animal models (Brewer, 2009).

Moreover, it must be considered that the largest amount of SOD1 extracted from SOD mice is in a copper-free form (apo-SOD) although intracellular concentration of copper is extremely higher compared to wild type animals (Tokuda et al., 2013). A copper chaperone (CCS) protein is known to specifically mediate copper ion-binding to SOD1 in the cytoplasm (Wong et al., 2000), however, iperexpression of the CCS in G93A SOD1 mice accelerates neurological deficits, and mice die within 2 weeks after birth (Son et al., 2009). By contrast, oral administration of diacetylbis(N(4)-methylthiosemicarbazonato copper (II) [Cu^II^(atsm)], a molecule with low toxicity, able to deliver copper in the CNS within minutes, prevents early mortality of G93A SOD1 mice over-expressing CCS protein (Williams et al., 2016). Interestingly, Cu^II^(atsm) administration to G93A symptomatic SOD1 mice delays onset of paralysis and extends lifespan (Son et al., 2009; McAllum et al., 2013).

**Figure 3:**
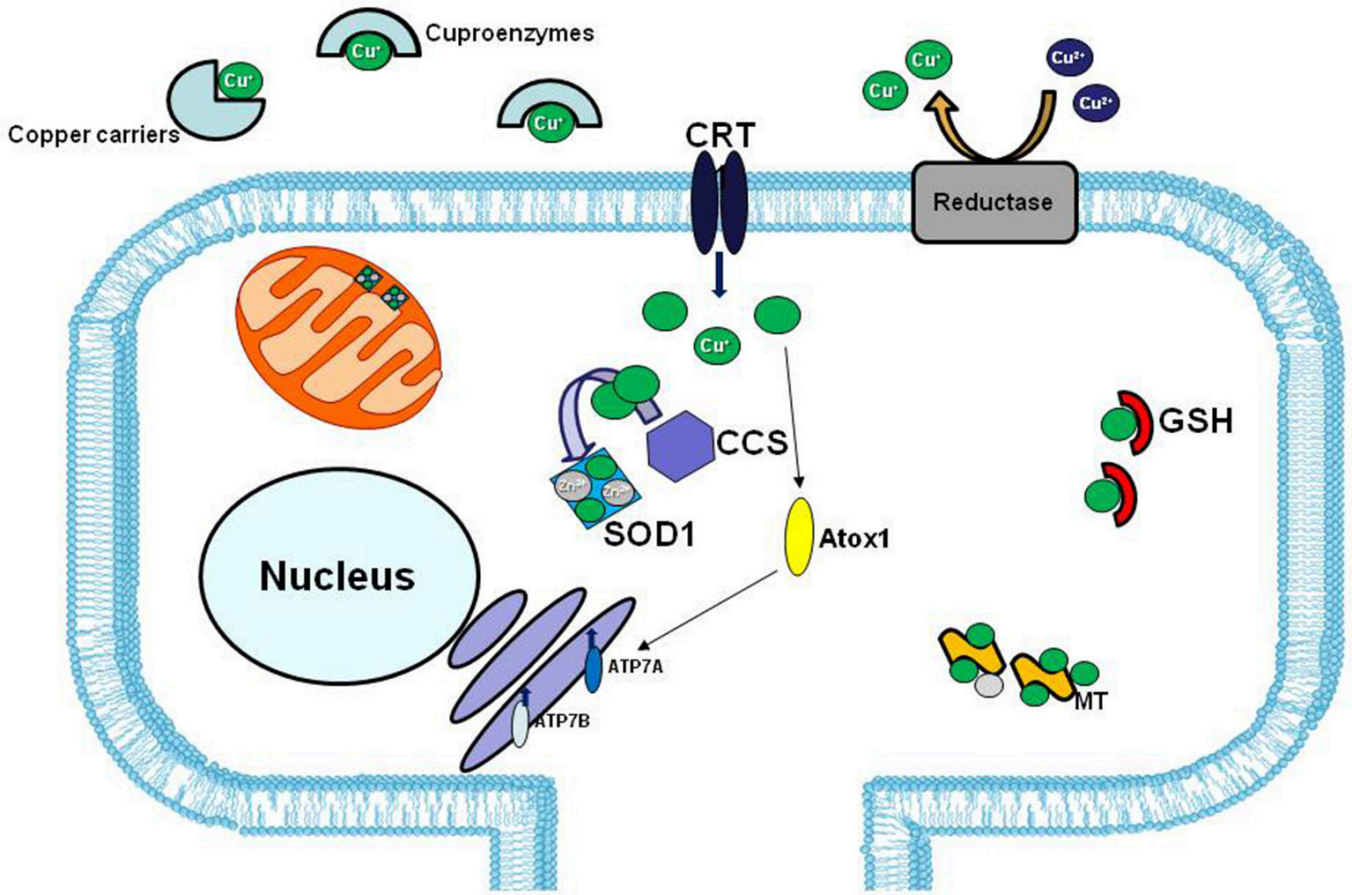
Copper distribution in the cell. In extracellular matrix copper (green balls) is bound to specific carriers or to enzymes that use copper as cofactor (cuproenzymes). The high affinity copper uptake protein 1 (CRT1) located on the plasma membrane, lets the copper enter inside the cell. Copper chaperons proteins, CCS and Atox1, facilitate copper loading on SOD1 and Cu-ATPases 7A and 7B, respectively. The two major copper-sequestering antioxidants are glutathione (GSH) and metallothionein (MT).

However, in human ALS cases, the involvement of copper dyshomeostasis on ALS etiology remains to be elucidate. In fact, although in the spinal cord of SOD1 transgenic line the copper chaperone CCS, that specifically delivers copper to SOD1, is commonly associated with mutated SOD1 in the neuronal Lewy body-like inclusions, this association is less common in human cases (Kato et al., 2001; Watanabe et al., 2001) (Figure 3).

## Role of zinc (Zn)

Zinc is an ubiquitous trace element that displays physiological roles being a catalytic, structural, and regulatory component in about 3000 known human metalloproteins, such as gene transcription factors and metalloenzymes (Maret, 2017); zinc also acts as intracellular second messenger and cell-cell signaling mediator in a large number of biological processes such as cellular proliferation, differentiation, migration, and apoptosis (Vallee and Falchuk, 1993; Franklin and Costello, 2009; Maret, 2013). Total cellular zinc concentration is estimated to reach about 200-300 μM, whereas free cytosolic zinc levels are maintained in the picomolar range (Maret, 2015, 2017; Portbury and Adlard, 2017).

Zinc is highly present in CNS either free or bound to metalloproteins. In particular, high levels of free chelatable Zn^2+^ are present into synaptic vesicles of a subpopulation of glutamatergic neurons (“zinc-containing” or “zincergic” neurons) of cerebral cortex, hippocampus and amygdala (Frederickson et al., 1983, 2000; Frederickson and Moncrieff, 1994), and into GABAergic terminals of the spinal cord (Wang et al., 2001). Upon synaptic activation vesicular zinc is released (Assaf and Chung, 1984) and modulates the activity of a variety of postsynaptic receptors ion channels and glutamate receptors (Li et al., 2001).

During normal physiological activity, the intracellular Zn^2+^ concentration is highly controlled by the concerted activity of membrane zinc transporters (ZnT and ZIP) and zinc binding proteins such as metallothioneins (MT) (Sekler et al., 2007; Kambe et al., 2015).

On the other hand, when an abnormal amount of zinc is released from presynaptic terminals or accumulated intracellularly after excessive release from MT, mitochondria and lysosomes, zinc rise can result in neuronal injury (Weiss et al., 2000). Several evidence demonstrates that alterations of zinc levels play a role in acute pathological conditions, including epileptic seizures and transient global cerebral ischemia (Koh et al., 1996; Weiss et al., 2000; Shuttleworth and Weiss, 2011), as well as in chronic neuropathologies, including Alzheimer's disease, Parkinson's disease, multiple sclerosis and amyotrophic lateral sclerosis (ALS) (Frederickson et al., 2005; Sensi et al., 2009; Szewczyk, 2013; Choi et al., 2017).

An abundant zinc-containing enzyme is SOD1, which binds one zinc atom per subunit, transferred to each nascent SOD1 monomer before the copper binding. Although zinc does not participate to enzymatic catalysis and wild-type SOD1 affinity for zinc is ~7,000-fold weaker than for copper, zinc coordination with amino acid residues in the active site is crucial for structural stability and proper functioning of SOD1 (Rakhit and Chakrabartty, 2006). Moreover, the correct and complete zinc binding plays a key role in the regulation of SOD1 folding and indirectly affects the catalytic activities of SOD1, accelerating the folding reaction. On the other hand, zinc-deficient SOD1 displays a propensity to misfold and self-aggregate in toxic amyloid-like species. Mutant SOD1 is less stable and is likely to become zinc-deficient and to aggregate (Roberts et al., 2007; Sirangelo and Iannuzzi, 2017). Likewise, wild-type enzyme aggregates *in vivo* if undermetalated; thus suggesting that not only SOD1 mutations themselves but the reduced zinc binding or zinc dissociation can play a crucial role in the familiar and sporadic forms of ALS.

Although the mechanisms responsible of copper and zinc dyshomeostasis are still unclear, it has been proposed that the impairment of zinc homeostasis could be a crucial event in ALS pathogenesis. Indeed, in two different studies, the analyses of zinc content in the cerebrospinal fluid from individuals with sporadic ALS disease and age-matched control subjects show a significant increase of zinc levels in ALS patients (Kanias and Kapaki, 1997; Hozumi et al., 2011). Moreover, among ALS patients, a correlation has been found between sex and zinc serum levels; in fact, male patients show higher serum levels than female ones (Kanias and Kapaki, 1997).

Furthermore, studies in G93A transgenic mice model of ALS, which overexpress mutant human G93A mutation in the SOD1 gene, confirm that zinc dyshomeostasis may contribute to the pathogenesis of this form of fALS. Indeed, Kim et al. (2009) observe that the appearance of ALS signs is accompanied by the presence of numerous spinal degenerating motor neurons and astrocytes in the spinal cords of G93A SOD1 transgenic mice that accumulate zinc. Moreover, they show that zinc elevation in these cells induces lipid peroxidation, as demonstrated by increased production of 4-hydroxy-2,3-nonenal (HNE), which itself contributes to disrupt Zn^2+^ homeostasis by trigger zinc release from MT and from G93A SOD1 (Kim et al., 2009).

ALS onset and progression depend on various interplaying processes that together lead to degeneration and atrophy of motor neurons. Zinc dyshomeostasis or zinc accumulation and oxidative injury may be important contributors. On the other hand, G93A SOD1 showed a weaker affinity for zinc and zinc depleted SOD1 may also contribute to neurodegenerative process inducing nitrosactive stress with peroxynitrite and catalyzes nitration of protein tyrosine residues (Goto et al., 2000; Puttaparthi et al., 2002).

Similarly, zinc and copper distribution in the spinal cord of G93A and other SOD1 transgenic mice, carrying H46R/H48Q, G37R human SOD1 mutations is altered in white matter (Lelie et al., 2011).

Experimental data about zinc distribution in white and gray matter in sporadic ALS patients are less clear and sometimes contradictory compared to those obtained in transgenic models. In fact, Tomik et al. (2006) found that zinc markedly increases in motor neurons, whereas lower levels are detected in the surrounding white matter (Tomik et al., 2006).

Altered expression of zinc binding proteins such as MT and membrane zinc transporters may also contribute to zinc dyshomeostasis. MT's main functional role is to sequester and/or dispense zinc contributing to zinc homeostasis. Of the three forms expressed in the murine nervous system, MT-I and MT-II are present in glial cells, whereas MT-III is a neuronal isoform. Metallothioneins are involved in the regulation of zinc availability within the cells, because they serve as zinc chaperones for accepting proteins during metalloenzymes synthesis, as apo-SOD1 (Suzuki and Kuroda, 1995). Changes in MT expression levels may promote mutant SOD1-induced toxicity; the reduction of MT buffering of zinc released from mutant SOD1 can contribute to intracellular zinc elevation. Interestingly, MT-I/II and MT-III levels are significantly reduced in the spinal cords of sporadic ALS patients and precedes the degeneration of MNs (Hozumi et al., 2008). In particular, MT-III levels are reduced in the gray matter of the lumbar spinal cord in the late phase of ALS (Hozumi et al., 2008) and MT-III deletion significantly reduces G93A SOD1 mice survival causing a pronounced loss of MNs and, in turn, accelerating the decline of motor functions (Puttaparthi et al., 2002). More, the increase of MT-III expression in the lumbar spinal cord of G93A SOD1 transgenic mice is able to prevent the loss of MNs in ALS model mice and to prolong the life span (Hashimoto et al., 2011).

Furthermore, also the removal of non-neuronal metallothionein, MT-I/II, may alter motor functions in ALS. In fact, MT-I/II deletion causes a decline in motor functions without an apparent severe neuronal loss because, even if motor neurons are present, they may be dysfunctional due to astrogliosis and microgliosis (Puttaparthi et al., 2002).

Membrane zinc transporters finely control intracellular zinc concentrations, regulating zinc uptake and efflux, sequestration and release across biological membranes. Two families of zinc transporter proteins have been identified, the Zinc Transporters or ZnTs (ZnT1-10) and the Zrt-, Irt-related proteins or ZIP (ZIP 1-14), which move zinc ions from cytosol to extracellular space or lumens of intracellular compartments and from extracellular space and lumens to cytosol, respectively (Kambe et al., 2015). To date, it has been demonstrated that, among membrane zinc transporters, the expression of ZnT6, present in the secretory pathway where zinc is required for correct folding and assembly of proteins, is significantly decreased in the spinal cords of sporadic ALS patients and precedes the degeneration of (Kaneko et al., 2015).

Earlier studies by Homma et al. (2013) suggest that zinc depleted wild-type SOD1 is able to induce a conformational change that allow the interaction between SOD1 and Derlin-1, a component of ER-associated degradation (ERAD) machinery, resulting in Derlin-1 inhibition and, in turn, in a block of ERAD and ER stress response activation. Consequently, ER stress stimulates ZnT3 and ZnT6 expression to favor zinc influx in the secretory pathway in order to prevent the accumulation of misfolded proteins (Homma et al., 2013). Thus, the reduced expression of ZnT3 and ZnT6 may compromise zinc uptake in secretory pathway and, in turn, contribute to ER stress described in ALS (Ito et al., 2009).

**Figure 4:**
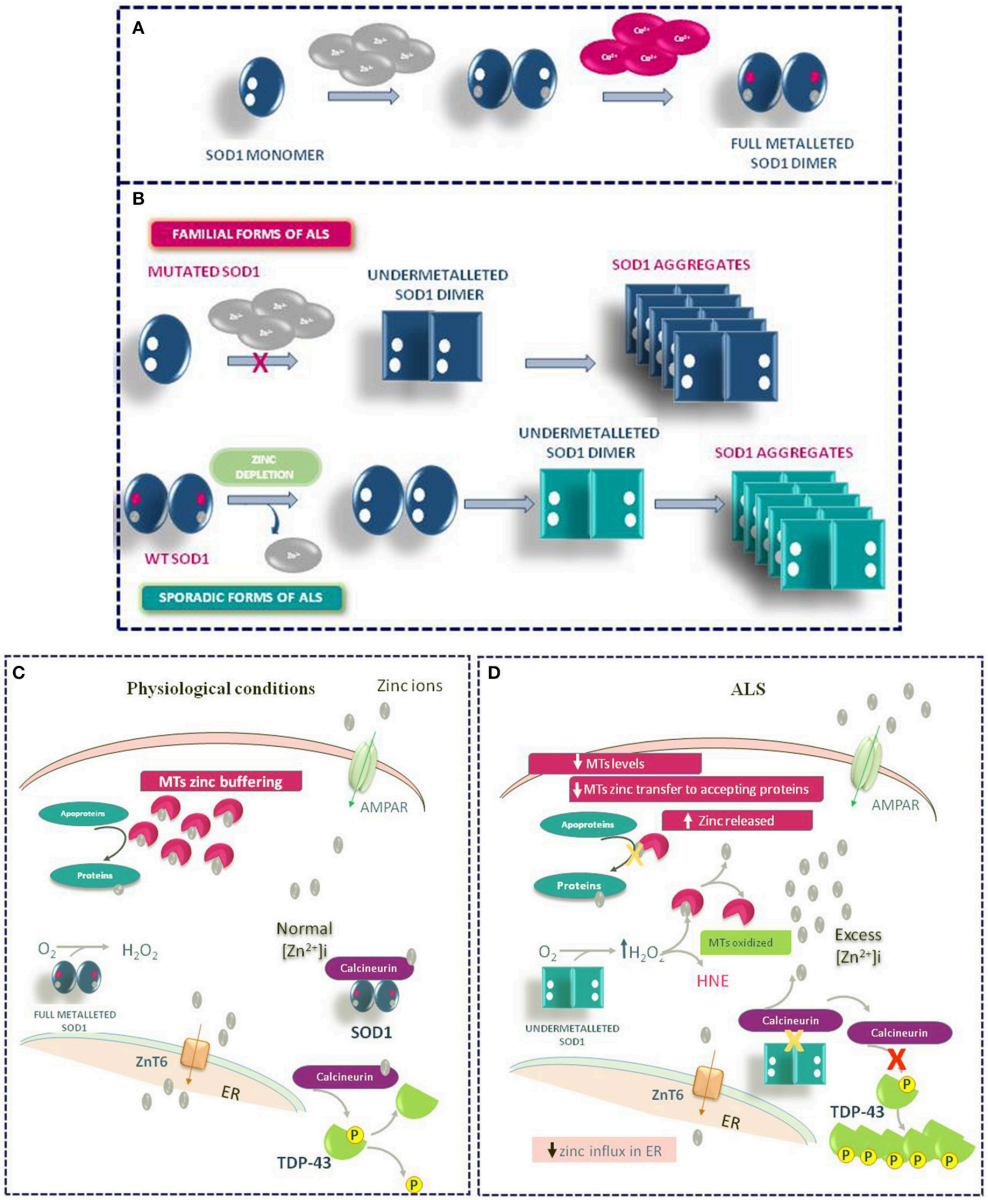
Role of Zinc in ALS Pathophysiology. **(A)** Physiologic mechanism of SOD1 dimer formation. **(B)** Mechanism of SOD1 aggregate formation in familiar and sporadic forms of ALS. **(C)** General physiological mechanism of Zinc homeostasis in motor neurons. **(D)** Pathophysiological mechanism of TDP-43 aggregation occurring in ALS under Zinc homeostasis de-regulation.

Other mechanisms may contribute to the Zn dyshomeostasis due to increased susceptibility of mutated SOD1 to lose zinc in ALS. In the intracellular milieu, SOD1 interacts with other proteins. Agbas e co-authors demonstrate that the metallophosphatase calcineurin interacts with SOD1 and this interaction is necessary for calcineurin activation (Agbas et al., 2007). Partial inactivation of calcineurin occurs in both sporadic and familiar ALS patients as well as in an asymptomatic carriers of a dominant SOD1 mutation (Ferri et al., 2004). Kim et al. (2018) demonstrate in rodent models of ALS that the reduced calcineurin activity is linked to a weaker interaction of mutated G93A SOD1 with calcineurin. Consequently, the failure of this interaction may create local metal toxicity; in fact, since the SOD1–calcineurin interaction is impaired, zinc ions would be dissociated from both calcineurin and SOD1. Interestingly, the authors found a significant increase of zinc concentration in the lumbar spinal cord of G93A SOD1 rats and mice. On the other hand, the cervical, thoracic, and sacral regions of transgenic rodents did not achieve significant levels of free zinc, though greater than wild type levels of label zinc (Kim et al., 2018). Moreover, the compromised activity of calcineurin may explain the presence of hyperphosphorylated TDP-43 protein aggregates in the spinal cord of ALS animal models (Kim et al., 2018) (Figure 4).

The hypothesis that excitotoxicity may contribute to MN degeneration in ALS is supported by the fact that till now the only drug able to slow the progression of ALS, riluzole, is an inhibitor of glutamate release (Doble, 1996). In ALS, rather than NMDA glutamate receptors, AMPA/kainate receptors may play a role allowing excessive zinc accumulation in MNs. In particular, injury is linked to the activation of a subset of AMPA/kainate receptors, Ca^2+^/Zn^2+^ permeable AMPA, highly expressed in these neuronal populations (Carriedo et al., 1996). Indeed, while the disease progression is rapid in SOD1 mutant mice expressing high levels of Ca-AMPA channels in MNs, it is attenuated when the numbers of these channels is reduced (Tateno et al., 2004; Kuner et al., 2005).

## Role of iron (Fe)

Iron represents an essential metal for life, since it has a key role as cofactor of enzymes involved in several metabolic processes like DNA, RNA, and protein synthesis, and mitochondrial oxidation reactions. Nevertheless, since it is a redox ion, it can produce free radicals which, in the absence of appropriate defense mechanisms, may cause cell damage. Solid evidence produced in the last decades showed that mutations of genes encoding proteins involved in iron homeostasis are associated with degeneration of CNS cells (Ponka, 2004; Zecca et al., 2004). Indeed, alteration of iron homeostasis is involved in neuronal cell death also in ALS. In fact, oxidative stress (Barber et al., 2006) induces cell injury by disrupting cellular iron balance (Blasco et al., 2011), thus leading to a vicious circle. The effectiveness of iron chelation as therapeutic strategy in ALS mouse models support the main role of iron in the pathogenesis of ALS (Kasarskis et al., 1995; Jeong et al., 2009; Kupershmidt et al., 2009).

Different pathogenetic mechanisms have been proposed to explain the abnormal accumulation of iron in neurons and in glia observed in ALS mice. Among them: (1) alterations of proteins involved in both iron influx and sensing of intracellular iron concentrations; (2) blockage of anterograde axonal transport with a consequent iron accumulation in ventral motor neurons; and (3) increased mitochondrial iron load in neurons and glia leading to neurodegeneration (Jeong et al., 2009).

## Putative targets

The absence of effective therapeutics for ALS treatment together with recent findings here reviewed on the metal ion dis-homeostasis as crucial event in this fatal neurodegenerative disease have led to test new beneficial strategies based on the control of metal ion concentrations within the different cell types interested by the disease.

In fact, starting from the therapeutic benefits observed in SOD1 transgenic mice exposed to diacetyl-bis(4-methylthiosemicarbazonato)copperII [CuII(atsm)], a metal complex which provides Cu to the mutant protein and decreases the abundance of Cu-deficient SOD1 in treated mice at the spinal cord level (Roberts et al., 2014), it has been used a CuII(atsm) analog, ZnII(atsm), in order to establish if the zinc delivery to SOD1 could have beneficial effects, McAllum et al. have demonstrated the therapeutic outcome of ZnII(atsm) as confirmed by improvement of motor function and increase survival rate of the mice. These positive effects of ZnII(atsm) depends also on its ability to determine an augmented Cu content of the mutant SOD1 (McAllum et al., 2015). These results prove that ZnII(atsm) could be useful to develop therapeutic agents able to transmetallate with copper and increase its binding to Cu/Zn dismutase.

As demostrated by Anzilotti et al. (2018) another pharmacological target to be considered as putative approach to slow down ALS progression is NCX3, an isoform of the plasmamembrane protein Na^+^/Ca^2+^ exchanger. In fact, when this antiporter is overexpressed or pharmacologically activated, it is able to mitigate MNs degeneration observed in ALS through a reduction of the ionic imbalance occurring during the progression of this aggravating condition.

Since the excessive accumulation of iron ions into the brain can determine a series of deleterious effects such as the increase of oxidative stress, the overexpression of the proteins responsible for neuronal degeneration with final effects that culminate in the increased neuronal vulnerability, another possible strategy to be considered as valid pharmacological tool in patients with ALS could be the use of compounds able to control iron homeostasis (Matrone et al., 2004; Romney et al., 2011). This strategy could be more promising for those compounds able to overcome the BBB, thus penetrating into the brain parenchyma (Smith et al., 1991; Rogers and Lahiri, 2004). Indeed, the administration of the iron chelator desferrioxamine (DFO), can slow dementia progression in AD patients (Rogers and Lahiri, 2004) and could be useful in ALS patients too. However, this chelator is not stable in blood circulation showing difficulties to cross the BBB barrier (Bandyopadhyay et al., 2010). Differently from DFO, the Clioquinol (CQ), a trifunctional chelating agent of iron, copper and zinc ions, shows an excellent ability to cross the BBB, and, therefore, after systemic administration, it can localize in the brain, reducing significantly the cognitive deficit in patients affected by Alzheimer's Disease (Ritchie et al., 2003). However, a limitation on the use of CQ therapy is represented by the fact that its use has been clinically associated with the onset of myelinopathies (Zhang et al., 2013). Other putative druggable targets involved in the control of iron homeostasis are two regulatory proteins that act as sensor of changes in iron concentration in the cytoplasm of duodenal epithelial cells, where the absorption of food iron take place. These iron regulatory proteins are called IRP1 and IRP2. Binding of regulatory proteins, IRPs to an iron response element sequence (IRE) at the level of untranslated region (UTR) of ferritin gene occurs if the concentration of alimentary iron is low. This effect results in an increase in transferrin RNA messenger expression which is, in turn, translated into ferritin protein that is able to rapidly transport iron ions into the blood, necessary in conditions of deficiency. Instead, when the concentration of food iron is high, this causes its binding to the IRP, leading to the dissociation of IRPs from IRE and to the reduction of the target transcript translation, i.e., transferrin. IRE sequences, able to favor the transcription of specific proteins such as transferrin, have been found in the 5′-UTR region of the amyloid precursor protein (APP) and in the α-synuclein (α-Syn) gene transcript. Therefore, under conditions of iron accumulation, due to the non-binding of IRP proteins to IRE sequences, the transcript levels of α-Syn, APP, and amyloid β-peptide are reduced and consequently the formation of neurofibrillary aggregates, characteristic of Alzheimer's disease, is reduced. Overall, all these observations led to the conclusion that the inhibition of APP and of α-Syn, due to the lack of interaction of the IRPs with the IRE sequences caused by the sequestration of iron occurring through selective chelating agents, constitutes a very valid therapy in the treatment of human neurodegenerative diseases.

Presently, an innovative pharmacological approach to neurodegenerative diseases includes the developing of two types of therapeutic agents able to modulate iron homeostasis: the first constituted by IRE chemical inhibitors and the second class represented by iron chelating agents.

Other possible targets in ALS pathology are represented by copper-modifying proteins. Indeed, in rodent models of ALS, it has been shown that the containment of high levels of copper ion abnormalities can be a potential pharmacological tool for the treatment of ALS. Interestingly, copper chelators such as D-penicillamine, trientine and tetrathiomolybdate (TMM) have been successfully tested in SOD1 G93A animals where they were able to delay the onset of the disease and to increase mice life span. These compounds have been approved in late fifties of last century for the treatment of Wilson disease, a pathology caused by an autosomal recessive mutation in the gene that encodes the transmembrane protein ATPase (ATP7B), a copper-dependent P-type ATPase. Clinical features of Wilson disease are the accumulation of copper ions in different body organs like in the liver and in the brain (Brewer et al., 2003; Ala et al., 2007). However, D-penicillamine effectiveness in ALS have been investigated in different clinical trials, where it showed very little improvement in disease progression (Bousser and Malier, 1979; Conradi et al., 1982). On the other hands, TMM might be a more promising strategy, seen that its positive effects were observed even when administered after disease onset in G93A SOD1 mice, and differently from D-penicillamine and trientine, TTM possesses a higher chelating selectivity for copper ions and is able to cross the blood brain barrier (McQuaid and Mason, 1991; Tokuda et al., 2008).

Copper intracellular levels could be also lowered by increasing MT expression. Indeed, these small cysteine-rich proteins have a very high affinity for heavy metals and particularly for copper ions (Hamer, 1986; Juárez-Rebollar et al., 2017). Interestingly, hyperexpression by adenovirus injection of the neuronal isoform III, MT-III (Palmiter et al., 1992), in G93A SOD1 mice at the onset of the pathology, protects MNs from degenerative damage and prolongs the survival of 2-week-old mice (Hashimoto et al., 2011). Furthermore, dexamethasone, a sintetic glucocorticoid, known to induce the expression of the glial isoforms MT-I and MT-II, significantly prolongs survival of 15 days and slows disease progression even if administered after symptoms onset in G93A SOD1 mice (Tokuda et al., 2015). Interestingly, dexamethasone effect is not present in MT-I/II knockout mice, indicating that addressing copper regulation can be a valuable strategy for ALS pathology and it might be worth setting up clinical trials.

The protein aggregation is another important neuropathological hallmark of ALS and precisely in order to counteract this aspect of the disease, Evans et al. (2016) tested the efficacy of 2-(2-hydroxyphenyl)-benzoxazole (HBX), to slow the onset and progression of ALS in G93A SOD1 mice. HBX is a thioflavin-like compound with metal chelating and anti-aggregation properties. In summary, this study demonstrate that dietary supplementation of HBX has a neuroprotective function in slowing down the appearance of the ALS phenotype in G93A mutant mice (Evans et al., 2016).

## Conclusions

Collectively, in the present review we summarized the recent data concerning the crucial role of several ions and redox metals in ALS pathogenesis (Table 1). In particular, it was emphasized how metals dyshomeostasis is reported to cause oxidative damage. In fact, the redox capacity of iron and copper are identified as prominent factors for neurodegeneration in this devastating disease. It is now well known the role of SOD1 mutation on copper dysregulation in ALS. In addition, has been published that the metal chelator therapy in animal models of ALS ameliorates neuronal degeneration and increased survival of mice. However, despite the numerous studies carried out so far, the main cause of this lethal neurodegenerative disease is still unknown and therefore requires further investigation in order to identify new strategies for diagnosis and treatment of this fatal neurological disease.

**Table 1:** List of main ions de-regulated in ALS.

**Ion**	**Alterations occurring in CNS cells interested by ALS**	**Molecular targets**	**References**
Ca^2+^	Increase of [Ca^2+^] in MNs	NCX3 Calreticulin Sigma 1-Receptor	Michalak et al., 2009; Tadić et al., 2017; Anzilotti et al., 2018
Na^+^	Increase of total Na^+^ currents	Nav Na^+^/K^+^ATPase	Ruegsegger et al., 2016; Jaiswal, 2017
K^+^	Decreased activity of inwardly rectifying K^+^ channel	Kir4	Bataveljić et al., 2012
Cu^+^	Accumulation of Cu^+^ in spinal cord	Cu^II^(atsm)	Tokuda et al., 2008; Roberts et al., 2014; Tokuda and Furukawa, 2016
Zn^2+^	Increase of Zn^2+^ levels	Zn^II^(atsm) Zn Transporter	McAllum et al., 2015; Tokuda and Furukawa, 2016
Fe^2+^	Increase of Fe^2+/3+^ levels	IRE IRP	Ritchie et al., 2003; Kupershmidt et al., 2009

*Putative druggable targets are indicated*.

## Author contributions

GP, RS, NB, VV, LA, SaA, and LC manuscript orgranization. OC, AV, PC, NG, SeA, PB, and CF study of the state of art.

### Conflict of interest statement

The authors declare that the research was conducted in the absence of any commercial or financial relationships that could be construed as a potential conflict of interest.

## References

[B1] Agbas A. Hui D. Wang X. Tek V. Zaidi A. Michaelis E. K. (2007). Activation of brain calcineurin (Cn) by Cu-Zn superoxide dismutase (SOD1) depends on direct SOD1-Cn protein interactions occurring *in vitro* and *in vivo*. Biochem. J. 405, 51–59. 10.1042/BJ20061202 17324120 PMC1925239

[B2] Ala A. Walker A. P. Ashkan K. Dooley J. S. Schilsky M. L. (2007). Wilson's disease. Lancet 369, 397–408. 10.1016/S0140-6736(07)60196-2 17276780

[B3] Alessandri-Haber N. Alcaraz G. Deleuze C. Jullien F. Manrique C. Couraud F.. (2002). Molecular determinants of emerging excitability in rat embryonic motoneurons. J. Physiol. 541, 25–39. 10.1113/jphysiol.2001.013371 12015418 PMC2290306

[B4] Alexianu M. E. Ho B. K. Mohamed A. H. La Bella V. Smith R. G. Appel S. H. (1994). The role of calcium-binding proteins in selective motoneuron vulnerability in amyotrophic lateral sclerosis. Ann. Neurol. 36, 846–858. 10.1002/ana.410360608 7998770

[B5] Annunziato L. Boscia F. Pignataro G. (2013). Ionic transporter activity in astrocytes, microglia, and oligodendrocytes during brain ischemia. J. Cereb. Blood. Flow. Metab. 33, 969–982. 10.1038/jcbfm.2013.44 23549380 PMC3705429

[B6] Annunziato L. Pignataro G. Boscia F. Sirabella R. Formisano L. Saggese M.. (2007). ncx1, ncx2, and ncx3 gene product expression and function in neuronal anoxia and brain ischemia. Ann. N.Y. Acad. Sci. 1099, 413–426. 10.1196/annals.1387.050 17446481

[B7] Annunziato L. Pignataro G. Di Renzo G. F. (2004). Pharmacology of brain Na^+^/Ca^2+^ exchanger: from molecular biology to therapeutic perspectives. Pharmacol. Rev. 56, 633–654. 10.1124/pr.56.4.5 15602012

[B8] Anzilotti S. Brancaccio P. Simeone G. Valsecchi V. Vinciguerra A. Secondo A.. (2018). Preconditioning, induced by sub-toxic dose of the neurotoxin L-BMAA, delays ALS progression in mice and prevents Na^+^/Ca^2+^ exchanger 3 downregulation. Cell Death Dis. 9:206. 10.1038/s41419-017-0227-9 29434186 PMC5833681

[B9] Assaf S. Y. Chung S. H. (1984). Release of endogenous Zn2+ from brain tissue during activity. Nature 308, 734–736. 10.1038/308734a0 6717566

[B10] Bandyopadhyay S. Huang X. Lahiri D. K. Rogers J. T. (2010). Novel drug targets based on metallobiology of Alzheimer's disease. Expert Opin. Ther. Targets 14, 1177–1197. 10.1517/14728222.2010.525352 20942746 PMC6677255

[B11] Barber S. C. Mead R. J. Shaw P. J. (2006). Oxidative stress in ALS: a mechanism of neurodegeneration and a therapeutic target. Biochim. Biophys. Acta 1762, 1051–1067. 10.1016/j.bbadis.2006.03.008 16713195

[B12] Basun H. Forssell L. G. Wetterberg L. Winblad B. (1991). Metals and trace elements in plasma and cerebrospinal fluid in normal aging and Alzheimer's disease. J. Neural. Transm. Park. Dis. Dement. Sect. 3, 231–258. 1772577

[B13] Bataveljić D. Nikoli L. Milosević M. Todorović N. Andjus P. R. (2012). Changes in the astrocytic aquaporin-4 and inwardly rectifying potassium channel expression in the brain of the amyotrophic lateral sclerosis SOD1(G93A) rat model. Glia 60, 1991–2003. 10.1002/glia.22414 22987392

[B14] Beckh S. Noda M. Lübbert H. Numa S. (1989). Differential regulation of three sodium channel messenger RNAs in the rat central nervous system during development. EMBO J. 8, 3611–3616. 10.1002/j.1460-2075.1989.tb08534.x 2555170 PMC402042

[B15] Blasco H. Vourc'h P. Nadjar Y. Ribourtout B. Gordon P. H. Guettard Y. O.. (2011). Association between divalent metal transport 1 encoding gene (SLC11A2) and disease duration in amyotrophic lateral sclerosis. J. Neurol. Sci. 303, 124–127. 10.1016/j.jns.2010.12.018 21276595

[B16] Boscia F. D'Avanzo C. Pannaccione A. Secondo A. Casamassa A. Formisano L.. (2012). Silencing or knocking out the Na(+)/Ca(2+) exchanger-3 (NCX3) impairs oligodendrocyte differentiation. Cell Death Differ. 19, 562–572. 10.1038/cdd.2011.125 21959935 PMC3307971

[B17] Bousser M. G. Malier M. (1979). Penicillamine in amyotrophic lateral sclerosis. Lancet 1:168. 10.1016/S0140-6736(79)90572-5 84200

[B18] Brewer G. J. (2009). The use of copper-lowering therapy with tetrathiomolybdate in medicine. Expert Opin. Investig. Drugs 18, 89–97. 10.1517/13543780802621859 19053885

[B19] Brewer G. J. Hedera P. Kluin K. J. Carlson M. Askari F. Dick R. B.. (2003). Treatment of Wilson disease with ammonium tetrathiomolybdate: III. Initial therapy in a total of 55 neurologically affected patients and follow-up with zinc therapy. Arch. Neurol. 60, 379–385. 10.1001/archneur.63.4.521 12633149

[B20] Brooks B. R. Miller R. G. Swash M. Munsat T. L. (2000). El Escorial revisited: revised criteria for the diagnosis of amyotrophic lateral sclerosis. Amyotroph. Lateral Scler. Other Motor Neuron Disord. 1, 293–299. 10.1080/146608200300079536 11464847

[B21] Carrí M. T. Ferri A. Cozzolino M. Calabrese L. Rotilio G. (2003). Neurodegeneration in amyotrophic lateral sclerosis: the role of oxidative stress and altered homeostasis of metals. Brain Res. Bull. 61, 365–374. 10.1016/S0361-9230(03)00179-5 12909279

[B22] Carriedo S. G. Yin H. Z. Weiss J. H. (1996). Motor neurons are selectively vulnerable to AMPA/kainate receptor-mediated injury *in vitro*. J. Neurosci. 16, 4069–4079. 10.1523/JNEUROSCI.16-13-04069.1996 8753869 PMC6578994

[B23] Casamassa A. La Rocca C. Sokolow S. Herchuelz A. Matarese G. Annunziato L.. (2016). Ncx3 gene ablation impairs oligodendrocyte precursor response and increases susceptibility to experimental autoimmune encephalomyelitis. Glia 64, 1124–1137. 10.1002/glia.22985 27120265

[B24] Catterall W. A. (2014). Structure and function of voltage-gated sodium channels at atomic resolution. Exp. Physiol. 99, 35–51. 10.1113/expphysiol.2013.071969 24097157 PMC3885250

[B25] Chen Q. Vazquez E. J. Moghaddas S. Hoppel C. L. Lesnefsky E. J. (2003). Production of reactive oxygen species by mitochondria: central role of complex III. J. Biol. Chem. 278, 36027–36031. 10.1074/jbc.M304854200 12840017

[B26] Chen-Izu Y. Shaw R. M. Pitt G. S. Yarov-Yarovoy V. Sack J. T. Abriel H.. (2015). Na+ channel function, regulation, structure, trafficking and sequestration. J. Physiol. 593, 1347–1360. 10.1113/jphysiol.2014.281428 25772290 PMC4376415

[B27] Choi B. Y. Jung J. W. Suh S. W. (2017). The Emerging role of zinc in the pathogenesis of multiple sclerosis. Int. J. Mol. Sci. 18:E2070. 10.3390/ijms18102070 28956834 PMC5666752

[B28] Conradi S. Ronnevi L. O. Nise G. Vesterberg O. (1982). Long-time penicillamine-treatment in amyotrophic lateral sclerosis with parallel determination of lead in blood, plasma and urine. Acta Neurol. Scand. 65, 203–211. 10.1111/j.1600-0404.1982.tb03078.x 7080805

[B29] Couratier P. Hugon J. Sindou P. Vallat J. M. Dumas M. (1993). Cell culture evidence for neuronal degeneration in amyotrophic lateral sclerosis being linked to glutamate AMPA/kainate receptors. Lancet 341, 265–268. 10.1016/0140-6736(93)92615-Z 8093916

[B30] Crichton R. R. Dexter D. T. Ward R. J. (2008). Metal based neurodegenerative diseases — from molecular mechanisms to therapeutic strategies. Coord. Chem. Rev. 252, 1189–1199. 10.1016/j.ccr.2007.10.019

[B31] Doble A. (1996). The pharmacology and mechanism of action of riluzole. Neurology 47(6 Suppl. 4), S233–S241. 10.1212/WNL.47.6_Suppl_4.233S 8959995

[B32] Evans T. M. Bhattacharya A. Shi Y. Qi W. Block T. J. Chaudhuri A.. (2016). Moderate modulation of disease in the G93A model of ALS by the compound 2-(2-hydroxyphenyl)-benzoxazole (HBX). Neurosci. Lett. 624, 1–7. 10.1016/j.neulet.2016.04.035 27138280

[B33] Ferrer I. Tuñón T. Serrano M. T. Casas R. Alcántara S. Zujar M. J.. (1993). Calbindin D-28k and parvalbumin immunoreactivity in the frontal cortex in patients with frontal lobe dementia of non-Alzheimer type associated with amyotrophic lateral sclerosis. J. Neurol. Neurosurg. Psychiatry 56, 257–261. 10.1136/jnnp.56.3.257 8459241 PMC1014857

[B34] Ferri A. Nencini M. Battistini S. Giannini F. Siciliano G. Casali C. (2004). Activity of protein phosphatase calcineurin is decreased in sporadic and familial amyotrophic lateral sclerosis patients. J. Neurochem. 90, 1237–1242. 10.1111/j.1471-4159.2004.02588.x 15312178

[B35] Franklin R. B. Costello L. C. (2009). The important role of the apoptotic effects of zinc in the development of cancers. J. Cell. Biochem. 106, 750–757. 10.1002/jcb.22049 19160419 PMC2727867

[B36] Frederickson C. J. Klitenick M. A. Manton W. I. Kirkpatrick J. B. (1983). Cytoarchitectonic distribution of zinc in the hippocampus of man and the rat. Brain Res. 273, 335–339. 10.1016/0006-8993(83)90858-2 6616240

[B37] Frederickson C. J. Koh J. Y. Bush A. I. (2005). The neurobiology of zinc in health and disease. Nat. Rev. Neurosci. 6, 449–462. 10.1038/nrn1671 15891778

[B38] Frederickson C. J. Moncrieff D. W. (1994). Zinc-containing neurons. Biol. Signals 3, 127–139. 10.1159/000109536 7531563

[B39] Frederickson C. J. Suh S. W. Silva D. Frederickson C. J. Thompson R. B. (2000). Importance of zinc in the central nervous system: the zinc-containing neuron. J. Nutr. 130(5S Suppl), 1471S–1483S. 10.1093/jn/130.5.1471S 10801962

[B40] Gilgun-Sherki Y. Melamed E. Offen D. (2001). Oxidative stress induced-neurodegenerative diseases: the need for antioxidants that penetrate the blood brain barrier. Neuropharmacology 40, 959–975. 10.1016/S0028-3908(01)00019-3 11406187

[B41] Goldin A. L. (1999). Diversity of mammalian voltage-gated sodium channels. Ann. N. Y. Acad. Sci. 868, 38–50. 10.1111/j.1749-6632.1999.tb11272.x 10414280

[B42] Gong Y. H. Elliott J. L. (2000). Metallothionein expression is altered in a transgenic murine model of familial amyotrophic lateral sclerosis. Exp. Neurol. 162, 27–36. 10.1006/exnr.2000.7323 10716886

[B43] Goto J. J. Zhu H. Sanchez R. J. Nersissian A. Gralla E. B. Valentine J. S.. (2000). Loss of *in vitro* metal ion binding specificity in mutant copper-zinc superoxide dismutases associated with familial amyotrophic lateral sclerosis. J. Biol. Chem. 275, 1007–1014. 10.1074/jbc.275.2.1007 10625639

[B44] Grosskreutz J. Van Den Bosch L. Keller B. U. (2010). Calcium dysregulation in amyotrophic lateral sclerosis. Cell Calcium 47, 165–174. 10.1016/j.ceca.2009.12.002 20116097

[B45] Halliwell B. (2006). Oxidative stress and neurodegeneration: where are we now? J. Neurochem. 97, 1634–1658. 10.1111/j.1471-4159.2006.03907.x 16805774

[B46] Hamer D. H. (1986). Metallothionein. Annu. Rev. Biochem. 55, 913–951. 10.1146/annurev.bi.55.070186.004405 3527054

[B47] Hashimoto K. Hayashi Y. Watabe K. Inuzuka T. Hozumi I. (2011). Metallothionein-III prevents neuronal death and prolongs life span in amyotrophic lateral sclerosis model mice. Neuroscience 189, 293–298. 10.1016/j.neuroscience.2011.05.034 21640795

[B48] Hilton J. B. White A. R. Crouch P. J. (2015). Metal-deficient SOD1 in amyotrophic lateral sclerosis. J. Mol. Med. 93, 481–487. 10.1007/s00109-015-1273-3 25754173 PMC4408375

[B49] Holland K. D. Kearney J. A. Glauser T. A. Buck G. Keddache M. Blankston J. R.. (2008). Mutation of sodium channel SCN3A in a patient with cryptogenic pediatric partial epilepsy. Neurosci. Lett. 433, 65–70. 10.1016/j.neulet.2007.12.064 18242854 PMC2423278

[B50] Homma K. Fujisawa T. Tsuburaya N. Yamaguchi N. Kadowaki H. Takeda K.. (2013). SOD1 as a molecular switch for initiating the homeostatic ER stress response under zinc deficiency. Mol. Cell. 52, 75–86. 10.1016/j.molcel.2013.08.038 24076220

[B51] Hozumi I. Hasegawa T. Honda A. Ozawa K. Hayashi Y. Hashimoto K.. (2011). Patterns of levels of biological metals in CSF differ among neurodegenerative diseases. J. Neurol. Sci. 303, 95–99. 10.1016/j.jns.2011.01.003 21292280

[B52] Hozumi I. Yamada M. Uchida Y. Ozawa K. Takahashi H. Inuzuka T. (2008). The expression of metallothioneins is diminished in the spinal cords of patients with sporadic ALS. Amyotroph. Lateral Scler. 9, 294–298. 10.1080/17482960801934312 18608104

[B53] Ito Y. Yamada M. Tanaka H. Aida K. Tsuruma K. Shimazawa M.. (2009). Involvement of CHOP, an ER-stress apoptotic mediator, in both human sporadic ALS and ALS model mice. Neurobiol. Dis. 36, 470–476. 10.1016/j.nbd.2009.08.013 19733664

[B54] Jaiswal M. K. (2013). Calcium, mitochondria, and the pathogenesis of ALS: the good, the bad, and the ugly. Front. Cell. Neurosci. 7:199. 10.3389/fncel.2013.00199 24198760 PMC3813898

[B55] Jaiswal M. K. (2017). Riluzole but not melatonin ameliorates acute motor neuron degeneration and moderately inhibits SOD1-mediated excitotoxicity induced disrupted mitochondrial Ca2+ signaling in amyotrophic lateral sclerosis. Front. Cell. Neurosci. 10:295 10.3389/fncel.2016.00295 28111541 PMC5216043

[B56] Jaiswal M. K. Keller B. U. (2009). Cu/Zn superoxide dismutase typical for familial amyotrophic lateral sclerosis increases the vulnerability of mitochondria and perturbs Ca^2+^ homeostasis in SOD1G93A mice. Mol. Pharmacol. 75, 478–489. 10.1124/mol.108.050831 19060114

[B57] Jeong S. Y. Rathore K. I. Schulz K. Ponka P. Arosio P. David S. (2009). Dysregulation of iron homeostasis in the CNS contributes to disease progression in a mouse model of amyotrophic lateral sclerosis. J. Neurosci. 29, 610–619. 10.1523/JNEUROSCI.5443-08.2009 19158288 PMC6665152

[B58] Jomova K. Valko M. (2011). Advances in metal-induced oxidative stress and human disease. Toxicology 283, 65–87. 10.1016/j.tox.2011.03.001 21414382

[B59] Juárez-Rebollar D. Rios C. Nava-Ruíz C. Méndez-Armenta M. (2017). Metallothionein in brain disorders. Oxid. Med. Cell. Longev. 2017:5828056 10.1155/2017/5828056 29085556 PMC5632493

[B60] Kambe T. Tsuji T. Hashimoto A. Itsumura N. (2015). The physiological, biochemical, and molecular roles of zinc transporters in zinc homeostasis and metabolism. Physiol. Rev. 95, 749–784. 10.1152/physrev.00035.2014 26084690

[B61] Kaneko M. Noguchi T. Ikegami S. Sakurai T. Kakita A. Toyoshima Y.. (2015). Zinc transporters ZnT3 and ZnT6 are downregulated in the spinal cords of patients with sporadic amyotrophic lateral sclerosis. J. Neurosci. Res. 93, 370–379. 10.1002/jnr.23491 25284286

[B62] Kanias G. D. Kapaki E. (1997). Trace elements, age, and sex in amyotrophic lateral sclerosis disease. Biol. Trace Elem. Res. 56, 187–201. 10.1007/BF02785392 9164664

[B63] Kasarskis E. J. Tandon L. Lovell M. A. Ehmann W. D. (1995). Aluminum, calcium, and iron in the spinal cord of patients with sporadic amyotrophic lateral sclerosis using laser microprobe mass spectroscopy: a preliminary study. J. Neurol. Sci. 130, 203–208. 10.1016/0022-510X(95)00037-3 8586987

[B64] Kato S. Sumi-Akamaru H. Fujimura H. Sakoda S. Kato M. Hirano A.. (2001). Copper chaperone for superoxide dismutase co-aggregates with superoxide dismutase 1 (SOD1) in neuronal Lewy body-like hyaline inclusions: an immunohistochemical study on familial amyotrophic lateral sclerosis with SOD1 gene mutation. Acta Neuropathol. 102, 233–238. 10.1007/s004010000355 11585247

[B65] Katsuno M. Tanaka F. Sobue G. (2012). Perspectives on molecular targeted therapies and clinical trials for neurodegenerative diseases. J. Neurol. Neurosurg. Psychiatry 83, 329–335. 10.1136/jnnp-2011-301307 22323772

[B66] Kim J. Kim T. Y. Hwang J. J. Lee J. Y. Shin J. H. Gwag B. J.. (2009). Accumulation of labile zinc in neurons and astrocytes in the spinal cords of G93A SOD-1 transgenic mice. Neurobiol. Dis. 34, 221–229. 10.1016/j.nbd.2009.01.004 19344646

[B67] Kim J. M. Billington E. Reyes A. Notarianni T. Sage J. Agbas E.. (2018). Impaired Cu-Zn superoxide dismutase (SOD1) and calcineurin (Cn) interaction in ALS: a presumed consequence for TDP-43 and zinc aggregation in Tg SOD1G93A rodent spinal cord tissue. Neurochem. Res. 10.1007/s11064-017-2461-z [Epub ahead of print]. 29299811 PMC6345727

[B68] Koh J. Y. Suh S. W. Gwag B. J. He Y. Y. Hsu C. Y. Choi D. W. (1996). The role of zinc in selective neuronal death after transient global cerebral ischemia. Science 272, 1013–1016. 10.1126/science.272.5264.1013 8638123

[B69] Kubat Öktem E. Mruk K. Chang J. Akin A. Kobertz W. R. Brown R. H. Jr. (2016). Mutant SOD1 protein increases Nav1.3 channel excitability. J. Biol. Phys. 42, 351–370. 10.1007/s10867-016-9411-x 27072680 PMC4942418

[B70] Kuner R. Groom A. J. Bresink I. Kornau H. C. Stefovska V. Müller G.. (2005). Late-onset motoneuron disease caused by a functionally modified AMPA receptor subunit. Proc. Natl. Acad. Sci. U.S.A. 102, 5826–5831. 10.1073/pnas.0501316102 15827116 PMC556301

[B71] Kupershmidt L. Weinreb O. Amit T. Mandel S. Carri M. T. Youdim M. B. (2009). Neuroprotective and neuritogenic activities of novel multimodal iron-chelating drugs in motor-neuron-like NSC-34 cells and transgenic mouse model of amyotrophic lateral sclerosis. FASEB J. 23, 3766–3779. 10.1096/fj.09-130047 19638399

[B72] Lampert A. Hains B. C. Waxman S. G. (2006). Upregulation of persistent and ramp sodium current in dorsal horn neurons after spinal cord injury. Exp. Brain Res. 174, 660–666. 10.1007/s00221-006-0511-x 16718433

[B73] Lanzillotta A. Pignataro G. Branca C. Cuomo O. Sarnico I. Benarese M.. (2013). Targeted acetylation of NF-kappaB/RelA and histones by epigenetic drugs reduces post-ischemic brain injury in mice with an extended therapeutic window. Neurobiol. Dis. 49, 177–189. 10.1016/j.nbd.2012.08.018 22971966

[B74] Lelie H. L. Liba A. Bourassa M. W. Chattopadhyay M. Chan P. K. Gralla E. B.. (2011). Copper and zinc metallation status of copper-zinc superoxide dismutase from amyotrophic lateral sclerosis transgenic mice. J. Biol. Chem. 286, 2795–2806. 10.1074/jbc.M110.186999 21068388 PMC3024775

[B75] Li Y. Hough C. J. Frederickson C. J. Sarvey J. M. (2001). Induction of mossy fiber –> Ca3 long-term potentiation requires translocation of synaptically released Zn2+. J. Neurosci. 21, 8015–8025. 10.1523/JNEUROSCI.21-20-08015.2001 11588174 PMC6763855

[B76] Lin C. L. Kong Q. Cuny G. D. Glicksman M. A. (2012). Glutamate transporter EAAT2: a new target for the treatment of neurodegenerative diseases. Future Med. Chem. 4, 1689–1700. 10.4155/fmc.12.122 22924507 PMC3580837

[B77] Lomen-Hoerth C. Murphy J. Langmore S. Kramer J. H. Olney R. K. Miller B. (2003). Are amyotrophic lateral sclerosis patients cognitively normal? Neurology 60, 1094–1097. 10.1212/01.WNL.0000055861.95202.8D 12682312

[B78] Lutsenko S. Bhattacharjee A. Hubbard A. L. (2010). Copper handling machinery of the brain. Metallomics 2, 596–608. 10.1039/c0mt00006j 21072351

[B79] Maret W. (2013). Zinc biochemistry: from a single zinc enzyme to a key element of life. Adv. Nutr. 4, 82–91. 10.3945/an.112.003038 23319127 PMC3648744

[B80] Maret W. (2015). Analyzing free zinc(II) ion concentrations in cell biology with fluorescent chelating molecules. Metallomics 7, 202–211. 10.1039/c4mt00230j 25362967

[B81] Maret W. (2017). Zinc in cellular regulation: the nature and significance of “zinc signals.” Int. J. Mol. Sci. 18:E2285 10.3390/ijms18112285 29088067 PMC5713255

[B82] Matrone C. Pignataro G. Molinaro P. Irace C. Scorziello A. Di Renzo G. F.. (2004). HIF-1α reveals a binding activity to the promoter of iNOS gene after permanent middle cerebral artery occlusion. J. Neurochem. 90, 368–378. 10.1111/j.1471-4159.2004.02483.x 15228594

[B83] McAllum E. J. Lim N. K. Hickey J. L. Paterson B. M. Donnelly P. S. Li Q. X.. (2013). Therapeutic effects of CuII(atsm) in the SOD1-G37R mouse model of amyotrophic lateral sclerosis. Amyotroph. Lateral Scler. Frontotemporal Degener. 14, 586–590. 10.3109/21678421.2013.824000 23952668

[B84] McAllum E. J. Roberts B. R. Hickey J. L. Dang T. N. Grubman A. Donnelly P. S.. (2015). Zn II(atsm) is protective in amyotrophic lateral sclerosis model mice via a copper delivery mechanism. Neurobiol. Dis. 81, 20–24. 10.1016/j.nbd.2015.02.023 25766674

[B85] McQuaid A. Mason J. (1991). A comparison of the effects of penicillamine, trientine, and trithiomolybdate on [35S]-labeled metallothionein *in vitro*; implications for Wilson's disease therapy. J. Inorg. Biochem. 41, 87–92. 10.1016/0162-0134(91)80002-Y 2033396

[B86] Michalak M. Groenendyk J. Szabo E. Gold L. I. Opas M. (2009). Calreticulin, a multi-process calcium-buffering chaperone of the endoplasmic reticulum. Biochem. J. 417, 651–666. 10.1042/BJ20081847 19133842

[B87] Molinaro P. Cataldi M. Cuomo O. Viggiano D. Pignataro G. Sirabella R.. (2013). Genetically modified mice as a strategy to unravel the role played by the Na(+)/Ca (2+) exchanger in brain ischemia and in spatial learning and memory deficits. Adv. Exp. Med. Biol. 961, 213–222. 10.1007/978-1-4614-4756-6_18 23224882

[B88] Molinaro P. Viggiano D. Nisticò R. Sirabella R. Secondo A. Boscia F.. (2011). Na^+^ -Ca2^+^ exchanger (NCX3) knock-out mice display an impairment in hippocampal long-term potentiation and spatial learning and memory. J. Neurosci. 31, 7312–7321. 10.1523/JNEUROSCI.6296-10.2011 21593315 PMC6622590

[B89] Mouhid Al-Achbili L. Moreno-Ortega A. J. Matías-Guiu J. Cano-Abad M. F. Ruiz-Nuño A. (2016). ITH33/IQM9.21 provides neuroprotection in a novel ALS model based on TDP-43 and Na+/Ca2+ overload induced by VTD. Neurosci. Lett. 633, 28–32. 10.1016/j.neulet.2016.09.009 27619542

[B90] Mühling T. Duda J. Weishaupt J. H. Ludolph A. C. Liss B. (2014). Elevated mRNA-levels of distinct mitochondrial and plasmamembrane Ca(2+) transporters in individual hypoglossal motor neurons of endstage SOD1 transgenic mice. Front. Cell. Neurosci. 8:353. 10.3389/fncel.2014.00353 25452714 PMC4231948

[B91] Palecek J. Lips M. B. Keller B. U. (1999). Calcium dynamics and buffering in motoneurones of the mouse spinal cord. J. Physiol. 520 (Pt 2), 485–502. 10.1111/j.1469-7793.1999.00485.x 10523417 PMC2269591

[B92] Palmiter R. D. Findley S. D. Whitmore T. E. Durnam D. M. (1992). MT-III, a brain-specific member of the metallothionein gene family. Proc. Natl. Acad. Sci. U.S.A. 89, 6333–6337. 10.1073/pnas.89.14.6333 1631128 PMC49495

[B93] Pannaccione A. Secondo A. Molinaro P. D'Avanzo C. Cantile M. Esposito A.. (2012). A new concept: Aβ1-42 generates a hyperfunctional proteolytic NCX3 fragment that delays caspase-12 activation and neuronal death. J. Neurosci. 32, 10609–10617. 10.1523/JNEUROSCI.6429-11.2012 22855810 PMC6621392

[B94] Park S. B. Kiernan M. C. Vucic S. (2017). Axonal excitability in amyotrophic lateral sclerosis: axonal excitability in ALS. Neurotherapeutics 14, 78–90. 10.1007/s13311-016-0492-9 27878516 PMC5233634

[B95] Petrozziello T. Secondo A. Tedeschi V. Esposito A. Sisalli M. Scorziello A. (2017). ApoSOD1 lacking dismutase activity neuroprotects motor neurons exposed to beta-methylamino-L-alanine through the Ca(^2+^)/Akt/ERK1/2 prosurvival pathway. Cell Death Differ. 24, 511–522. 10.1038/cdd.2016.154 28085149 PMC5344211

[B96] Plaitakis A. Caroscio J. T. (1987). Abnormal glutamate metabolism in amyotrophic lateral sclerosis. Ann. Neurol. 22, 575–579. 10.1002/ana.410220503 2892463

[B97] Ponka P. (2004). Hereditary causes of disturbed iron homeostasis in the central nervous system. Ann. N. Y. Acad. Sci. 1012, 267–281. 10.1196/annals.1306.022 15105272

[B98] Portbury S. D. Adlard P. A. (2017). Zinc signal in brain diseases. Int. J. Mol. Sci. 18:E2506. 10.3390/ijms18122506 29168792 PMC5751109

[B99] Puttaparthi K. Gitomer W. L. Krishnan U. Son M. Rajendran B. Elliott J. L. (2002). Disease progression in a transgenic model of familial amyotrophic lateral sclerosis is dependent on both neuronal and non-neuronal zinc binding proteins. J. Neurosci. 22, 8790–8796. 10.1523/JNEUROSCI.22-20-08790.2002 12388585 PMC6757708

[B100] Que E. L. Domaille D. W. Chang C. J. (2008). Metals in neurobiology: probing their chemistry and biology with molecular imaging. Chem. Rev. 108, 1517–1549. 10.1021/cr078203u 18426241

[B101] Rakhit R. Chakrabartty A. (2006). Structure, folding, and misfolding of Cu,Zn superoxide dismutase in amyotrophic lateral sclerosis. Biochim. Biophys. Acta 1762, 1025–1037. 10.1016/j.bbadis.2006.05.004 16814528

[B102] Ringholz G. M. Appel S. H. Bradshaw M. Cooke N. A. Mosnik D. M. Schulz P. E. (2005). Prevalence and patterns of cognitive impairment in sporadic ALS. Neurology 65, 586–590. 10.1212/01.wnl.0000172911.39167.b6 16116120

[B103] Ritchie C. W. Bush A. I. Mackinnon A. Macfarlane S. Mastwyk M. MacGregor L.. (2003). Metal-protein attenuation with iodochlorhydroxyquin (clioquinol) targeting Abeta amyloid deposition and toxicity in Alzheimer disease: a pilot phase 2 clinical trial. Arch. Neurol. 60, 1685–1691. 10.1001/archneur.60.12.1685 14676042

[B104] Roberts B. R. Lim N. K. McAllum E. J. Donnelly P. S. Hare D. J. Doble P. A.. (2014). Oral treatment with Cu(II)(atsm) increases mutant SOD1 *in vivo* but protects motor neurons and improves the phenotype of a transgenic mouse model of amyotrophic lateral sclerosis. J. Neurosci. 34, 8021–8031. 10.1523/JNEUROSCI.4196-13.2014 24899723 PMC6608261

[B105] Roberts B. R. Tainer J. A. Getzoff E. D. Malencik D. A. Anderson S. R. Bomben V. C.. (2007). Structural characterization of zinc-deficient human superoxide dismutase and implications for ALS. J. Mol. Biol. 373, 877–890. 10.1016/j.jmb.2007.07.043 17888947 PMC2175016

[B106] Rogers J. T. Lahiri D. K. (2004). Metal and inflammatory targets for Alzheimer's disease. Curr. Drug Targets 5, 535–551. 10.2174/1389450043345272 15270200

[B107] Romney S. J. Newman B. S. Thacker C. Leibold E. A. (2011). HIF-1 regulates iron homeostasis in Caenorhabditis elegans by activation and inhibition of genes involved in iron uptake and storage. PLoS Genet. 7:e1002394. 10.1371/journal.pgen.1002394 22194696 PMC3240588

[B108] Rothstein J. D. Martin L. J. Kuncl R. W. (1992). Decreased glutamate transport by the brain and spinal cord in amyotrophic lateral sclerosis. N. Engl. J. Med. 326, 1464–1468. 10.1056/NEJM199205283262204 1349424

[B109] Rothstein J. D. Van Kammen M. Levey A. I. Martin L. J. Kuncl R. W. (1995). Selective loss of glial glutamate transporter GLT-1 in amyotrophic lateral sclerosis. Ann. Neurol. 38, 73–84. 10.1002/ana.410380114 7611729

[B110] Ruegsegger C. Maharjan N. Goswami A. Filézac de L'Etang A. Weis J. Troost D.. (2016). Aberrant association of misfolded SOD1 with Na(+)/K(+)ATPase-α3 impairs its activity and contributes to motor neuron vulnerability in ALS. Acta Neuropathol. 131, 427–451. 10.1007/s00401-015-1510-4 26619836

[B111] Rusina R. Ridzon P. Kulist'ák P. Keller O. Bartos A. Buncová M. (2010). Relationship between ALS and the degree of cognitive impairment, markers of neurodegeneration and predictors for poor outcome. A prospective study. Eur. J. Neurol. 17, 23–30. 10.1111/j.1468-1331.2009.02717.x 19572947

[B112] Saberi S. Stauffer J. E. Schulte D. J. Ravits J. (2015). Neuropathology of amyotrophic lateral sclerosis and its variants. Neurol. Clin. 33, 855–876. 10.1016/j.ncl.2015.07.012 26515626 PMC4628785

[B113] Sekler I. Sensi S. L. Hershfinkel M. Silverman W. F. (2007). Mechanism and regulation of cellular zinc transport. Mol. Med. 13, 337–343. 10.2119/2007-00037.Sekler 17622322 PMC1952664

[B114] Sensi S. L. Paoletti P. Bush A. I. Sekler I. (2009). Zinc in the physiology and pathology of the CNS. Nat. Rev. Neurosci. 10, 780–791. 10.1038/nrn2734 19826435

[B115] Sheykhansari S. Kozielski K. Bill J. Sitti M. Gemmati D. Zamboni P.. (2018). Redox metals homeostasis in multiple sclerosis and amyotrophic lateral sclerosis: a review. Cell Death Dis. 9:348. 10.1038/s41419-018-0379-2 29497049 PMC5832817

[B116] Shuttleworth C. W. Weiss J. H. (2011). Zinc: new clues to diverse roles in brain ischemia. Trends Pharmacol. Sci. 32, 480–486. 10.1016/j.tips.2011.04.001 21621864 PMC3148334

[B117] Siklós L. Engelhardt J. I. Alexianu M. E. Gurney M. E. Siddique T. Appel S. H. (1998). Intracellular calcium parallels motoneuron degeneration in SOD-1 mutant mice. J. Neuropathol. Exp. Neurol. 57, 571–587. 10.1097/00005072-199806000-00005 9630237

[B118] Sirabella R. Secondo A. Pannaccione A. Scorziello A. Valsecchi V. Adornetto A.. (2009). Anoxia-induced NF-kappaB-dependent pregulation of NCX1 contributes to Ca2+ refilling into endoplasmic reticulum in cortical neurons. Stroke 40, 922–929. 10.1161/STROKEAHA.108.531962 19164785

[B119] Sirangelo I. Iannuzzi C. (2017). The role of metal binding in the amyotrophic lateral sclerosis-related aggregation of copper-zinc superoxide dismutase. Molecules 22:E1429. 10.3390/molecules22091429 28850080 PMC6151412

[B120] Smith C. D. Carney J. M. Starke-Reed P. E. Oliver C. N. Stadtman E. R. Floyd R. A.. (1991). Excess brain protein oxidation and enzyme dysfunction in normal aging and in Alzheimer disease. Proc. Natl. Acad. Sci. U.S.A. 88, 10540–10543. 10.1073/pnas.88.23.10540 1683703 PMC52964

[B121] Sokolow S. Manto M. Gailly P. Molg,ó J. Vandebrouck C. Vanderwinden J. M.. (2004). Impaired neuromuscular transmission and skeletal muscle fiber necrosis in mice lacking Na/Ca exchanger 3. J. Clin. Invest. 113, 265–273. 10.1172/JCI18688 14722618 PMC310749

[B122] Son M. Fu Q. Puttaparthi K. Matthews C. M. Elliott J. L. (2009). Redox susceptibility of SOD1 mutants is associated with the differential response to CCS over-expression *in vivo*. Neurobiol. Dis. 34, 155–162. 10.1016/j.nbd.2009.01.005 19320055 PMC2835407

[B123] Speisky H. Gómez M. Burgos-Bravo F. López-Alarcón C. Jullian C. Olea-Azar C.. (2009). Generation of superoxide radicals by copper-glutathione complexes: redox-consequences associated with their interaction with reduced glutathione. Bioorg. Med. Chem. 17, 1803–1810. 10.1016/j.bmc.2009.01.069 19230679

[B124] Suzuki K. T. Kuroda T. (1995). Transfer of copper and zinc from ionic and metallothionein-bound forms to Cu, Zn–superoxide dismutase. Res. Commun. Mol. Pathol. Pharmacol. 87, 287–296. 7620821

[B125] Szewczyk B. (2013). Zinc homeostasis and neurodegenerative disorders. Front. Aging Neurosci. 5:33. 10.3389/fnagi.2013.00033 23882214 PMC3715721

[B126] Tadić V. Malci A. Goldhammer N. Stubendorff B. Sengupta S Prell T.. (2017). Sigma 1 receptor activation modifies intracellular calcium exchange in the G93AhSOD1 ALS model. Neuroscience 359, 105–118. 10.1016/j.neuroscience.2017.07.012 28723387

[B127] Tateno M. Sadakata H. Tanaka M. Itohara S. Shin R. M. Miura M.. (2004). Calcium-permeable AMPA receptors promote misfolding of mutant SOD1 protein and development of amyotrophic lateral sclerosis in a transgenic mouse model. Hum. Mol. Genet. 13, 2183–2196. 10.1093/hmg/ddh246 15294873

[B128] Tokuda E. Furukawa Y. (2016). Copper homeostasis as a therapeutic target in amyotrophic lateral sclerosis with SOD1 mutations. Int. J. Mol. Sci. 17:E636. 10.3390/ijms17050636 27136532 PMC4881462

[B129] Tokuda E. Okawa E. Ono S. (2009). Dysregulation of intracellular copper trafficking pathway in a mouse model of mutant copper/zinc superoxide dismutase-linked familial amyotrophic lateral sclerosis. J. Neurochem. 111, 181–191. 10.1111/j.1471-4159.2009.06310.x 19656261

[B130] Tokuda E. Okawa E. Watanabe S. Ono S. Marklund S. L. (2013). Dysregulation of intracellular copper homeostasis is common to transgenic mice expressing human mutant superoxide dismutase-1s regardless of their copper-binding abilities. Neurobiol. Dis. 54, 308–319. 10.1016/j.nbd.2013.01.001 23321002

[B131] Tokuda E. Ono S. Ishige K. Watanabe S. Okawa E. Ito Y.. (2008). Ammonium tetrathiomolybdate delays onset, prolongs survival, and slows progression of disease in a mouse model for amyotrophic lateral sclerosis. Exp. Neurol. 213, 122–128. 10.1016/j.expneurol.2008.05.011 18617166

[B132] Tokuda E. Watanabe S. Okawa E. Ono S. (2015). Regulation of intracellular copper by induction of endogenous metallothioneins improves the disease course in a mouse model of amyotrophic lateral sclerosis. Neurotherapeutics 12, 461–476. 10.1007/s13311-015-0346-x 25761970 PMC4404437

[B133] Tomik B. Chwiej J. Szczerbowska-Boruchowska M. Lankosz M. Wójcik S. Adamek D.. (2006). Implementation of X-ray fluorescence microscopy for investigation of elemental abnormalities in amyotrophic lateral sclerosis. Neurochem. Res. 31, 321–331. 10.1007/s11064-005-9030-6 16733809

[B134] Trotti D. Aoki M. Pasinelli P. Berger U. V. Danbolt N. C. Brown R. H. Jr. (2001). Amyotrophic lateral sclerosis-linked glutamate transporter mutant has impaired glutamate clearance capacity. J. Biol. Chem. 276, 576–582. 10.1074/jbc.M003779200 11031254

[B135] Trotti D. Rolfs A. Danbolt N. C. Brown R. H. Jr. Hediger M. A. (1999). SOD1 mutants linked to amyotrophic lateral sclerosis selectively inactivate a glial glutamate transporter. Nat. Neurosci. 2:848 10.1038/8091 10461226

[B136] Valko M. Jomova K. Rhodes C. J. Kuča K. Musílek K. (2016). Redox- and non-redox-metal-induced formation of free radicals and their role in human disease. Arch. Toxicol. 90, 1–37. 10.1007/s00204-015-1579-5 26343967

[B137] Vallee B. L. Falchuk K. H. (1993). The biochemical basis of zinc physiology. Physiol. Rev. 73, 79–118. 10.1152/physrev.1993.73.1.79 8419966

[B138] Visconti A. Cotichini R. Cannoni S. Bocca B. Forte G. Ghazaryan A.. (2005). Concentration of elements in serum of patients affected by multiple sclerosis with first demyelinating episode: a six-month longitudinal follow-up study. Ann. Ist. Super. Sanita. 41, 217–222. 16244396

[B139] von Lewinski F. Keller B. U. (2005). Ca2+, mitochondria and selective motoneuron vulnerability: implications for ALS. Trends Neurosci. 28, 494–500. 10.1016/j.tins.2005.07.001 16026864

[B140] Wang Z. Li J. Y. Dahlström A. Danscher G. (2001). Zinc-enriched GABAergic terminals in mouse spinal cord. Brain Res. 921, 165–172. 10.1016/S0006-8993(01)03114-6 11720723

[B141] Watanabe M. Dykes-Hoberg M. Culotta V. C. Price D. L. Wong P. C. Rothstein J. D. (2001). Histological evidence of protein aggregation in mutant SOD1 transgenic mice and in amyotrophic lateral sclerosis neural tissues. Neurobiol. Dis. 8, 933–941. 10.1006/nbdi.2001.0443 11741389

[B142] Weiss J. H. Sensi S. L. Koh J. Y. (2000). Zn(2+): a novel ionic mediator of neural injury in brain disease. Trends Pharmacol. Sci. 21, 395–401. 10.1016/S0165-6147(00)01541-8 11050320

[B143] Wen X. Westergard T. Pasinelli P. Trotti D. (2017). Pathogenic determinants and mechanisms of ALS/FTD linked to hexanucleotide repeat expansions in the C9orf72 gene. Neurosci. Lett. 636, 16–26. 10.1016/j.neulet.2016.09.007 27619540 PMC5148671

[B144] Williams J. R. Trias E. Beilby P. R. Lopez N. I. Labut E. M. Bradford C. S.. (2016). Copper delivery to the CNS by CuATSM effectively treats motor neuron disease in SOD(G93A) mice co-expressing the Copper-Chaperone-for-SOD. Neurobiol. Dis. 89, 1–9. 10.1016/j.nbd.2016.01.020 26826269 PMC4785045

[B145] Wong P. C. Waggoner D. Subramaniam J. R. Tessarollo L. Bartnikas T. B. Culotta V. C.. (2000). Copper chaperone for superoxide dismutase is essential to activate mammalian Cu/Zn superoxide dismutase. Proc. Natl. Acad. Sci. U.S.A. 97, 2886–2891. 10.1073/pnas.040461197 10694572 PMC16025

[B146] Zecca L. Youdim M. B. Riederer P. Connor J. R. Crichton R. R. (2004). Iron, brain ageing and neurodegenerative disorders. Nat. Rev. Neurosci. 5, 863–873. 10.1038/nrn1537 15496864

[B147] Zhang Y. H. Raymick J. Sarkar S. Lahiri D. K. Ray B. Holtzman D.. (2013). Efficacy and toxicity of clioquinol treatment and A-beta42 inoculation in the APP/PSI mouse model of Alzheimer's disease. Curr. Alzheimer Res. 10, 494–506. 10.2174/1567205011310050005 23627708

